# Adaptive Load Forecasting Methodology Based on Generalized Additive Model with Automatic Variable Selection

**DOI:** 10.3390/s22197247

**Published:** 2022-09-24

**Authors:** Sovjetka Krstonijević

**Affiliations:** 1 Institute Mihajlo Pupin, University of Belgrade, 11000 Belgrade, Serbia; sovjetka.krstonijevic@pupin.rs; 2 School of Electrical Engineering, University of Belgrade, 11000 Belgrade, Serbia

**Keywords:** electricity load forecasting, generalized additive model, automatic variable selection, smart grid

## Abstract

For decentralized energy management in a smart grid, there is a need for electric load forecasting at different places in the grid hierarchy and for different levels of aggregation. Load forecasting functionality relies on the load time series prediction model, which provides accurate forecasts. Complex and heterogeneous multi-source load time series in a smart grid require flexible modeling approaches to meet the accuracy demand. This work proposes an adaptive load forecasting methodology based on the generalized additive model (GAM) with the big data estimation method. It is based on a set of GAM terms, constructed for a specific multi-source load forecasting application in the grid and a procedure that dynamically selects the most relevant terms and generates forecasts for particular load time series. Data from publicly available New York Independent System Operator (NYISO) databases are used for testing. The 24-hour-ahead forecasting results for eleven New York City zones, of different sizes and types, indicate the applicability of the proposed methodology.

## 1. Introduction

An accurate electricity load forecast is the main prerequisite for various activities in the grid that enables its stability, reliability, and operating cost reduction, hence the quality of electricity supply. This information is important for the operation of many applications and services, as well as for all those in charge of timely grid management, planning, and electricity market optimization. The supported decisions and applications rely on forecasts for different time horizons that could be from minutes to years ahead, categorized into very-short-, short-, medium-, and long-term forecasting functionality. For most of daily tasks in the grid, such as production scheduling, economic load dispatch, demand side management (DSM), peak load reduction, etc., the forecasting horizon from the next hour to a day ahead is of interest, which is the domain of short-term load forecasting (STLF).

The increased penetration of renewable energy sources (RESs), integration of new loads such as, e.g., electric vehicles, and new energy market mechanisms introduce significant variability and uncertainty to both the supply and demand side in the power system, imposing the need for its management at the local level. Access to local energy systems and different load aggregations enables better integration of RESs, the implementation of DSM programs and other electricity market products, and services and general grid optimization. Such circumstances change the specification of STLF forecasting tools developed for centralized management, typically for a single, system load time series. The main challenges in developing local forecasting functionality could be summarized as follows:

- Local load time series, produced by a smaller number of consumers, generally have more complex profiles, requiring advanced modeling techniques for achieving satisfactory forecasting accuracy.
- A higher level of automation and adaptation to data and over time is required for supporting local load forecasting functionality. The energy management in the smart grid implies operation with multiple energy sources, and the forecasting tool has to cope with many heterogeneous load time series and their changes, which requires versatile models and adaptive methodologies.
- The load forecasting functionality evolves to a more general predictive tool, and the interpretability of the implemented model has recently been emphasized as useful for, e.g., the exceptional events’ forecasts, creating tariffs, energy purchasing, etc.

Achieving satisfactory load forecasting accuracy is, by itself, a demanding task due to multi-seasonality, the nonlinear dependency of externals, and the nonstationarity of load time series. The new challenges are in the complexity and diversity of the load profiles at the different places in the grid hierarchy. The traditional approaches, based on multivariable linear regression (MLR) or time series (TS) models, i.e., ARMA and its variants, verified, mainly, for a higher level of aggregations, are, also, in use for smart-meter-based forecasting [1] or for energy management in buildings [2]. Simple and fast algorithms make them favorable solutions for real-time forecasting functionalities, as for, e.g., dynamic demand response, presented in [3]. The improvement in modeling the nonlinearities in the load, resulting in better forecasting accuracy, has been obtained with the machine learning (ML) and artificial intelligence (AI) techniques. An often used approach is based on support vector machine (SVM) and examples of SVM-based load forecasting could be found, either, for the system and distribution level [4] or for office buildings [5]. Random forest (RF) is a computationally inexpensive technique, which is used for the load prediction of the whole country load [6], but also for diverse types of building clusters [7]. Artificial neural networks (ANNs) are the most popular ML-based load forecasting approach, widely accepted and applied for the overall system [8], substations [9], buildings [10], and consumers [11]. With the emergence of deep neural networks (DNNs), their application to the STLF problem became available, having a better perspective to capture the load profiles with higher accuracy. In [12], the DNN-based STLF methodology, tested on 40 industrial customers, outperformed the shallow NN and classical TS models in the accuracy of forecasts. Recently, the recurrent neural networks (RNNs) have gained attention, as they are suitable for modeling sequential data such as time series. In particular, long short-term memory (LSTM) networks, a type of RNN for long-term dependencies, produced better accuracy compared to several TS models in [13] and several benchmarks in [14]. A number of developed STLF approaches aim to take advantage of the favorable properties of two or more models, by their hybridization, e.g., ARIMA/NN in [15], used for the overall system and 18 Spanish sub-regions, or through the concepts of ensemble prediction, as in [16], where four prediction algorithms, i.e., the Holt–Winters model (HWT), sigma support vector regression (sSVR), seasonal ARIMA, and the State-Space Model (SSM), were fused in order to improve the forecasting accuracy and robustness for smart meter aggregates. In [17], to optimize the computational efficiency and forecasting accuracy, the authors used the ensemble empirical mode decomposition (EEMD) algorithm for the separation of the smooth and periodic low-frequency load component, modeled with the MLR, from high-frequency load variations, described by the LSTM.

Although various modeling techniques are capable of capturing local load profiles with satisfactory accuracy, for smart grid applications, it is necessary to evaluate their performances on larger load populations. Depending on the adopted modeling approach, the generalizability is achieved through relevant load features’ selection, the proper choice of model parameters, and/or hyperparameters. For example, in the mentioned paper [4], the authors developed an automatic SVR model-building procedure, using the particle swarm global optimization (PSG) technique for model parameter optimization and feature selection automation. Furthermore, in [7], auto-encoder (AE)-based feature extraction was proposed for the RF model and tested on several datasets for diverse types of building clusters. In [11], an optimal ANN architecture and set of model inputs, for 37 consumer groups, were obtained through multiple prediction simulations. In [18], the authors exploited the statistical properties of the load time series (i.e., correlation structure) to optimize the hyperparameters of the convolutional neural network (CNN) model. For building-level forecasting application, the RNN and CNN were used for the day-ahead load forecasting of three buildings in [19]. The same hyperparameters (layers and number of neurons) for all buildings and separate model parameters (weights and bias) were selected and tuned through the cross-validation approach. In order to avoid prior analysis and to automate the search of the optimal model structure, in [20], the authors proposed the GA-LSTM approach where the genetic algorithm (GA) was used to find the optimal inputs and the hidden neurons of the LSTM network, for particular data. Although new NN topologies dominate traditional approaches in forecasting accuracy, the computational burden and sensitivity to hyperparameters could limit their wider application. In practice, there is a need for often model updating and methodologies based on iterative parameter tuning (manually using cross-validation) or on slow optimizers (such as GA), which could be suboptimal.

In this work, a methodology based on the generalized additive model (GAM) [21] is proposed. The GAM is an interpretable ML technique, which extends the standard linear model framework with the nonparametric terms for modeling nonlinear relationships. The capability of the GAM to capture load profiles generated at the different places in the grid has been verified with good forecasting accuracy at the national [22], regional [23], and substation levels [24], buildings [25], and for small aggregates of consumer clusters [26]. The advantages of the GAM’s interpretability are presented in [25], on the example of monitoring and diagnosing activities in smart buildings. In the recently published paper [27], the GAM was coupled with the Kalman filters for quick adjustment to changes of electricity consumption caused by the COVID-19 lockdown in France. The important improvements of the standard GAM estimation procedure, up to big data methods, were presented in [28,29]. Based on these results, the GAM framework is used to develop an adaptive load forecasting method, projecting its general application in the grid. In this regard, the main contributions of this paper are as follows:

- An approach is presented for constructing a dictionary of GAM terms for a specific forecasting application in the grid. The terms standardize the main load features determined by the level of aggregation, forecast horizon, and available data. As an example, the dictionary for eleven New York Independent System Operator (NYISO) zones, for day-ahead load forecasting functionality and available data for loads and temperatures, was constructed and evaluated. Differences between NYISO zones in population density, weather conditions, rural/urban balance, and average consumption support testing the generalizability of the approach.
- A dynamic, shrinkage-type term selection, embedded into the fast GAM estimation procedure, is developed. It automatically and continuously removes uninformative terms from the dictionary, serving as a mechanism for its dynamic adaptation to a specific load.
- The ability of the proposed methodology to describe all load time series from the NYISO dataset and the predictive performances for each zone are evaluated and improvements recommended.
- Promising results of 24-hour-ahead forecasts, for real working conditions, were obtained.

The paper is organized as follows: Section 2 summarizes the basics of the GAM framework used in this work. Section 3 describes a way to define GAM terms for a given load forecasting application. Section 4 details the results, while Section 5 gives the conclusions and future research directions.

## 2. Methodology

The proposed methodology relies on an adopted class of the GAM structure and the automatic variable selection method, developed for additive models, presented in the next few subsections.

### 2.1. GAM Structure

A generalized additive model, GAM covers a wide range of structures, and the “prototype” of the one considered in this paper has the form:

(1) $$y=\sum_{i}\beta_{0i}x_{i}+\sum_{j}f_{j}\left(x_{j}\right)+\sum_{k}D_{k}f_{k}\left(x_{k}\right)+\sum_{l}f_{l}\left(x_{l1},x_{l2}\right)+e$$

where variable *y* (or its transformation) is represented with the sum of several types of GAM terms, which are functions of the input variables, i.e., features, $x_{i}$, $x_{k}$, $x_{l1}$, $x_{l1}$. Each term is constructed separately and implements the effect that the input variable or combination of variables produces on *y*. The type of GAM term is determined by its functional form, depending on whether it models a linear or nonlinear effect of one or more variables. $\beta_{0i}x_{i}$ is a standard linear model term, for modeling the linear effect of $x_{i}$. $f_{j}$s, $f_{k}$s, and $f_{l}$s are the smooth, unspecified functions of one or more variables, i.e., smoothers, used for representing nonlinearities in the model. There are no prior assumptions about the shape of the smoother, which is a fully data-driven, nonparametric term. The $D_{k}f_{k}\left(x_{k}\right)$ term models the smoother $f_{k}$ of $x_{k}$ whose shape depends on some other known variable, $D_{k}$. Of interest, here, is the case where $D_{k}$ is a categorical variable, where $f_{k}$ is assumed to have a different shape for each of the categories. In more specific cases, when $D_{k}$ is a binary variable, the presence ($D_{k}$ = 1) or absence ($D_{k}$ = 0) of smoother $f_{k}$ in the model is coded. $f_{l}$ is a smooth interaction term, derived from the bivariate function of $x_{l1}$ and $x_{l2}$, which models their mutual effect on *y*.

GAM estimation is based on regression techniques, and for this purpose, the smoothers in Model (1) are realized with the splines. Accordingly, a one-variable smoother ($f_{j}$ and $f_{k}$) of a general form, $f_{x}\left(x\right)$, is approximated by a linear combination of *q* known basis functions, $b_{i}\left(x\right)$, put in the columns of matrix $B_{x}$, and corresponding coefficients, $\beta_{i}$ from vector $\beta_{x}$, as:

(2) $$f_{x}\left(x\right)=\sum_{i=1}^{q}b_{i}\left(x\right)\beta_{i}=B_{x}\beta_{x}$$

The smooth interaction term of two variables, $x_{1}$ and $x_{2}$, is constructed using the tensor product of the bases $b_{1,i_{1}}\left(x_{1}\right)$ and $b_{2,i_{2}}\left(x_{2}\right)$, which belong to their individual spline representations, $f_{x_{1}}\left(x_{1}\right)$ and $f_{x_{2}}\left(x_{2}\right)$, according to:

(3) $$f\left(x_{1},x_{2}\right)=\sum_{i_{1}=1}^{q_{1}}\sum_{i_{2}=1}^{q_{2}}b_{1,i_{1}}\left(x_{1}\right)b_{2,i_{2}}\left(x_{2}\right)\beta_{i_{1}i_{2}}$$

The set of bases in the sum above implicitly contains the individual representations for $f_{x_{1}}\left(x_{1}\right)$ and $f_{x_{2}}\left(x_{2}\right)$ and a pure interaction, $f_{x_{1}x_{2}}\left(x_{1},x_{2}\right)$, which exclusively describes their mutual effect. To extract the basis representing only the interaction, the tensor product is decomposed, according to the procedure described in [21]. Then, the smooth interaction term is represented with the extracted basis, $B_{x_{1}x_{2}}$, and corresponding coefficients, $\beta_{x_{1}x_{2}}$, as $B_{x_{1}x_{2}}\beta_{x_{1}x_{2}}$.

The choice and specification of splines used for a smoother representation determine its modeling flexibility. For efficient approximation, the number of spline functions, *q*, has to be sufficient as it determines the smoother’s capacity to capture the nonlinearity of the models. Regarding the type of splines used, different realizations are possible, and they could be combined in the same model. Here, a cyclic cubic regression spline, CCRS, and a thin plate regression spline, TPRS, were used [21]. The CCRS spline is suitable for modeling cyclic nonlinearities. It uses cubic polynomials as basis functions to approximate segments of nonlinearity, joined at so-called knots. For cyclic nonlinearity, as, for example, weekly load seasonality, the knots placement is usually put equidistantly, for each a day of a week. For noncyclic nonlinearities, a common solution is a thin plate regression spline, TPRS, which is a general-purpose spline of any number of variables. The TPRS avoids knot placement consideration by using eigen decomposition for the automatic generation of *q*, the most informative basis for the given data.

The GAM terms are estimated simultaneously. By spline expansion, the GAM structure from (1) takes the form of a linear model, Y≈Bβ, where the set of observations of the variable being modeled $Y={\left[y_{1}\;y_{2}\ldots \right]}^{T}$ is represented with the model matrix $B=[X\ldots B_{x_{j}}\ldots B_{x_{k}}\ldots B_{x_{l1},x_{l2}}\ldots ]$, containing all linear terms, X, and evaluated basis functions $B_{x_{j}}$, $B_{x_{k}}$ and $B_{x_{l1},x_{l2}}$ for the $f_{j}$, $f_{k}$, and $f_{l}$ smoothers. $\beta ={\left[\beta_{0}\ldots \beta_{j}\ldots \beta_{k}\ldots \beta_{x_{l1},x_{l2}}\ldots \right]}^{T}$ is a vector of corresponding model coefficients that need to be calculated. The model is estimated by the penalized regression method, where the penalization controls the “wiggliness” of the smoothers. For any smoother in the model, *f*, represented with the spline coefficients β, its “wiggliness” is expressed by the functional $\beta^{T}S\beta$, where S is the known penalty matrix, specific to the type of spline used. For the interaction terms, a separate such penalty is associated with each variable. Combined with the associated parameter λ for smoothness control, the penalties are added to model the likelihood, l(β), to estimate the model by:

(4) $$\hat{\beta }=\underset{x}{\mathrm{argmax}}\;\{l\left(\beta \right)-\frac{1}{2}\sum_{j}\lambda_{j}\beta_{j}^{T}S_{j}\beta_{j}\}$$

For solving the above optimization problem, a penalized iteratively reweighted least squares (PIRLS) was chosen, while smoothing parameters were automatically selected by the restricted maximum likelihood (REML) [21]. The coefficients were estimated by iterating the PIRLS/REML as in [28,29].

### 2.2. Automatic Variable Selection

A key step in GAM specification is the proper choice of the input variables and corresponding terms so as to obtain the desired model performances. The variable selection (VS) procedure in the GAM searches a pool of initially nominated model terms and selects those that, for particular data, meet the specified model selection criteria. For practical multi-source data application, where the same set of GAM terms is evaluated for different data, the VS search needs to be automated.

In papers with the GAM-based load forecasting models, the VS is, usually, performed through numerous iterations, controlled by, for example, the results of residual checking [22] or forecasting accuracy [23]. Iterative search could be automated using some searching scheme (subset selection, stepwise procedures), controlled with the criteria, such as the Akaike information criterion (AIC), the Bayesian information criterion (BIC), or through cross-validation (CV). However, as the number of input variables increases, the searching becomes more time-consuming and computationally demanding.

An alternative method is shrinkage, which has good stability and predictive accuracy and a low computational cost. In the GAM, shrinkage additionally penalizes the estimation such that the model terms are “encouraged” to be shrunk to have zero effect and significant GAM terms that “survive” are automatically obtained. An example of shrinkage use for additive models is presented in [30], where the authors proposed a two-stage procedure, combining the Group least absolute shrinkage and selection operator (LASSO) method for model selection and regular model estimation, with three different model selection criteria, i.e., BIC, AIC, and Generalized CV.

An approach where the shrinkage mechanism is embedded into the estimation procedure is considered. In this regard, several methods, designed for additive models, are offered, as for example the component selection shrinkage operator (COSSO), [31], sparse additive models (SpAMs) [32], and generalized additive model selection (GAMSEL) [33]. Here, the method, introduced by Marra and Wood in [34], is used, primarily because of its simplicity and its easy integration into the estimation. Its main property is that the terms are selected along with the model estimation, in a single step, outputting a single model. The authors reported high predictive ability, while the results were comparable to the best subset selection method, which searches for all possible combinations of variables.

Marra and Wood exploited the fact that any type of spline space could be decomposed into a “wiggly” component (range space) and the rest (null space). The penalized estimation (explained in the previous section) removes the wiggliness of the function and reduces the model complexity. However, to eliminate the term effect in the model, its null space has to be penalized. The authors proposed two such methods, and for more details, one can see the original paper [34]. In the double-penalty (DP) approach, the authors introduced an additional penalty for penalizing the null space. The null space was obtained by decomposing the penalty matrix $S_{j}$ of each smoother into the eigenspace according to:

(5) $$S_{j}=U_{j}\Lambda_{j}U_{j}^{T}$$

where $U_{j}$ and $\Lambda_{j}$ are an eigenvector and eigenvalue matrix. The spline basis space for which $\Lambda_{j}$ contains zeros belongs to the basis null space. The shrinkage penalty matrix is then produced as:

(6) $$S_{j}^{*}=U_{j}^{*}{U_{j}^{*}}^{T}$$

where $U_{j}^{*}$ is the matrix of the eigenvectors for the corresponding zeros eigenvalues of $\Lambda_{j}$. With this mechanism, the model estimation criterion (4) is corrected up to:

(7) $$\hat{\beta }=\underset{x}{\mathrm{argmax}}\;\{l\left(\beta \right)-\frac{1}{2}\sum_{j}\left(\lambda_{j}\beta_{j}^{T}S_{j}\beta_{j}+\lambda_{j}^{*}\beta_{j}^{T}S_{j}^{*}\beta_{j})\right\}$$

where additional shrinkage parameters $\lambda_{j}^{*}$ are to be estimated. The criteria from (7) is an objective of the regular GAM estimation procedure, now with twice as many smoothing parameters for each penalized term.

### 2.3. Forecasting Procedure

GAM application to the time series forecasting problem is exemplified with variable *y*, modeled as:

(8) $$y_{t}=gam\left(y_{t-1},y_{t-2},\ldots ,x_{t},x_{t-1},\ldots \right)+e_{t}$$

where *y* in *t* depends on its previous realizations at t−1, t−2, …, etc., due to intrinsic correlation and on current and lagged values of variables explaining the external factors, placed in vector x. gam denotes the adopted initial GAM structure, defined with the set of terms. Based on (8), the rolling procedure arises: with each new value of *y* and x, in any *t*, the most recent *N* observations of model inputs, including $y_{t}$ and $x_{t}$, are used by PIRLS/REML/DP for model retraining and variable reselection. For available values of externals for t+1, ${\hat{x}}_{t+1}$, either real values or forecasts, the generated $gam_{t}$ outputs the forecast ${\hat{y}}_{t+1}$ according to:

(9) $${\hat{y}}_{t+1}=gam_{t}\left(y_{t},y_{t-1},\ldots ,{\hat{x}}_{t+1},x_{t},\ldots \right)$$

Further forecasts, i.e., ${\hat{y}}_{t+2}$, …, can be obtained from (9), recursively, having a prognosis for external variables, ${\hat{x}}_{t+2}$, …, etc. Continuous updating with VS re-initialization allows the model to dynamically adapt to changes in the input data. The described procedure supports the forecasting engine, which automatically generates forecasts for all time series for which the initial set of terms in gam and specified training set size, N, are valid assumptions.

## 3. Load Forecasting Model Construction

For multi-source load forecasting, the initially constructed set of GAM terms implements features common to all load time series for which the forecasting functionality is being developed. As an example, in the next section, a dictionary of load GAM terms is proposed, constructed for a day-ahead load forecasting for several NYISO zones. Although prepared for a specific dataset, the proposed dictionary can, generally, be used in the grid for forecasting the load of the same level of aggregation and for the same specification of the forecast horizon, types of external variables, and the availability of historical data. Furthermore, it can be easily expanded with the additional terms for introducing new inputs into the model and/or adapted for other forecasting application in the grid.

### 3.1. NYISO Data

The New York Independent System Operator (NYISO) controls the territory spanning the entire state of New York, partitioned into eleven zones. They differ in geographical size and population, from the highly populated, urban NYC zone of the New York City area, then the large, but low populated Mohawk Valley and rural North zone, to geographically small zones, such as Millwood. The zones, the abbreviations, their consumption level, and the number of residents are listed in Table 1.

Load data and meteorological variables were collected from available databases on the official NYISO site [35]. The data consist of consumption measurements and daily maximal and minimal temperature values, for each zone. A particular convenience is that the NYISO set also contains the historical values of temperatures forecasts in addition to the historical values of their realizations, which allows testing for realistic working conditions.

### 3.2. Load GAM Terms’ Dictionary for NYISO Zones

The NYISO zones produce load profiles characteristic of higher levels of aggregation, since the least populated North has about 80,000 consumers. For constructing the GAM terms for all NYISO zones, some general properties of the aggregated load and its dependence on temperature were considered.

The calendar-based load factors produce characteristic patterns of annual, weekly, and daily profiles, so that, in each instant, the load level depends on its position in the day, week, and year. A separate model for instants that belong to each observed load in a day (typically, for hourly, half-hourly measurements) was adopted, denoted as a single load, $L_{t}$, for the *t*-th daily sample. Splitting the daily profile with the separate time series models enables better incorporation of the load factors’ daily variation, projecting better forecasting accuracy.

For aggregated loads, the cyclic weekly and yearly variations of a single load could be represented with the smooth cyclic patterns $f_{w}\left(week\right)$ and $f_{y}\left(year\right)$, defined for each day of the week, week, and each day of the year, year. The smoothers are realized using cyclic CRS, with 7 equidistant knots for the weekly and 12 knots for the yearly cycle. The interaction term, $f_{w,y}\left(week,year\right)$, for incorporating the effect of weekly profile changes over the year was also added.

The most important external load factors are related to the weather conditions. To quantify the human-perceived temperature and HVAC utilization, responsible for the weather-induced load effect, in addition to the air temperature, the wind speed, humidity, cloud cover, solar radiation, etc., were used. In practice, the choice of weather variables included in the model depends on the availability and quality of the relevant data. For some grids (i.e., some regions), the air temperature is reported as sufficient for obtaining satisfying STLF accuracy, not being improved by adding the other weather variables [15], while, for example, in [36], it was shown that for estimating the summer load extremes, the additional weather variables are needed.

**Figure 1:**
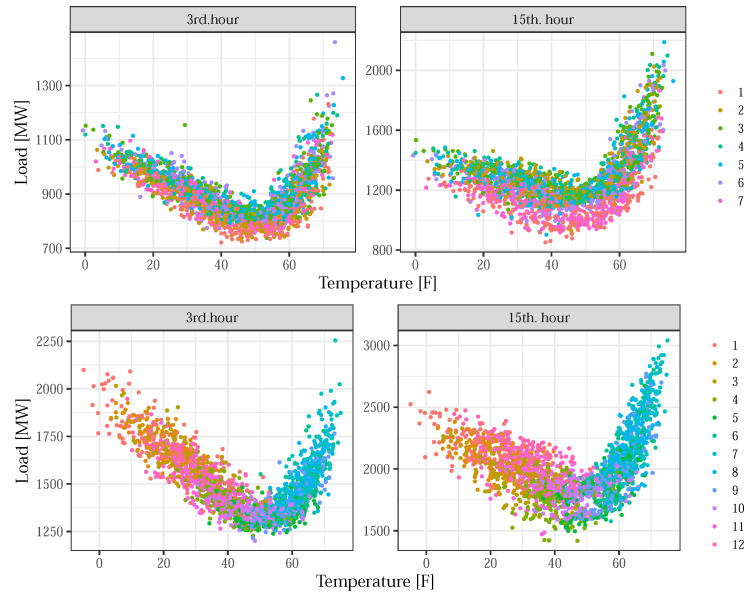
The differences in the load–temperature relationship between days of the week (**upper row**) and over the year (**bottom row**), for non-working (**left**) and working (**right**) hours.

Some properties of the load–temperature relationship are shown in Figure 1, using the available data. The scatter plots represent the average hourly consumption for typical non-working (left) and working (right) hours, versus average daily temperature. The differences in load response to temperature for different days of the week (upper row) and for different months within the year (bottom row) are highlighted. It is obvious that load–temperature relationship is driven by, more or less, different nonlinearities for different days of the week and times of the year, indicating the combined effect of temperature and basic calendar variables to the load.

For available daily maximum and minimum temperature values, $T_{mx}$ and $T_{mn}$, their combined effect on the load is fully represented with the separate terms $f_{Tmx}\left(T_{mx}\right)$, $f_{Tmn}\left(T_{mn}\right)$ and interaction $f_{T_{mx},T_{mn}}\left(T_{mx},T_{mn}\right)$. For weekly and yearly change adjustments, $f_{T_{x},week}\left(T_{x},week\right)$ and $f_{T_{x},year}\left(T_{x},year\right)$ are the interactions terms, where $T_{x}$ is the maximum/minimum temperature. With this set of terms, the overall daily weather-induced variation of the load is modeled. In order to account for the impact of the temperature history, i.e., the influence of the temperatures from previous days on current load, the same set of terms with the temperatures from the previous two days were included.

For STLF models trained on a multi-year dataset, a significant feature is the long-term variation in the consumption level, observed as an inter-annual trend. The economic factors, changes in population, industry growth, technology development, and other long-term factors are mentioned in the literature as the main causers of this effect. When not externally identified, a long-term trend is included in the model as a function of time (linear, polynomials, etc.) or in the GAM, with the dedicated spline, $f_{t}\left(trend\right)$.

The correlation in the load time series, not included with the basic profiles, is, typically, modeled with the historical load values from previous time instances, days, and weeks. To adjust a single load value, $L_{t}^{d}$, on a day *d* with the information from recent history, the loads from the entire previous week were added, with the splines $f_{1}\left(L_{t}^{d-1}\right)$, …, $f_{7}\left(L_{t}^{d-7}\right)$. In the same way, the loads from the two previous time instances, $f_{8}\left(L_{t-1}^{d}\right)$ and $f_{9}\left(L_{t-2}^{d}\right)$, were included, which is the way to account for the correlation between independently modeled single loads.

The departure from the regular load behavior is for so-called special days, a typical example being when the irregular behavior expected is for holidays. These irregularities are specific for each special day type (holidays), for each system or subsystem. In practice, special days are, usually, treated as a non-working day (Saturday/Sunday) or represented with the same model. Here, the special day effect is implemented by using the categorical variable, day type, DT, for coding four categories of forecasted days: a regular day that follows a regular day (default), holiday after regular day, regular day after holiday, and holiday after holiday. With DT in the model, the average changes in a single load level for these days are set, while with its interaction with the previous day’s load, DTx$f_{1}\left(L_{t}^{d-1}\right)$, these changes are, also, locally adjusted.

**Figure 2:**
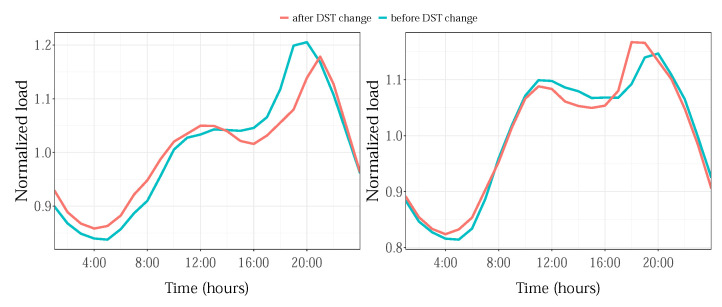
Daylight saving time effect for the year 2013, for the NYC zone.

Daylight saving time (DST) aims to make the most of day light in order to reduce the lighting electricity usage by moving the clock one hour forward in spring and one hour back in fall. For example, in the USA (for which we have testing data), DST lasts for 238 days, starting by skipping the second a.m. hour on the second Sunday of March and ends by doubling the second a.m. hour on the first Sunday of November. In load forecasting practice, the usual strategy is to interpolate loads for the missed morning 2nd hour in March with neighbor values for the 1st and 3rd hours and average loads for 2nd hour double occurrences in October, in order to enable the continuity of the 24-h daily profile in operation. However, the changes that the DST effect produces on the load profiles are rarely considered, and recently, in [37], it was shown that their inclusion in the model improves the forecasting accuracy. In Figure 2, the normalized load daily profiles for Sunday before and after the clock change in March (left) and in November (right) illustrate the daily profile deviations caused by DST.

Changes in the load caused by DST for different times in the day are related to the daylight period, which, for a certain region, depends on the time of the year. According to [37], it can be expected that the DST-induced changes are, also, specific for each day of the week. To include the DST load effect, we first adopted the daily binary variable, DS, set to 1 for all days between clock changes in March and October, while for other days, its value was 0. Next, we added a smooth, nonlinear difference to the yearly and weekly profiles with the terms DSx$f_{y}\left(year\right)$ and DSx$f_{w}\left(week\right)$, respectively.

The natural logarithm of the load was considered instead of its raw value, which has proven to be the most suitable transformation that gives the best results for the aggregated loads.

Summarizing the above, a dictionary of GAM load terms that describes a single load time series from the NYISO set is given in Table 2. The zonal STLF model is based on a set of such daily submodels for each observed load in the day.

## 4. Results and Discussion

The adequacy of the proposed GAM dictionary for describing all loads from the given set and the predictive performances of the generated zonal STLF models were first considered. The 24-hour-ahead forecasting, for real working conditions, was observed. The results, obtained for different seasons and for special days, are highlighted. The influence of the included daylight saving time effect on the accuracy of the forecast was also observed.

### 4.1. Data Preparation

The load and temperature data for the period from 1 January 2009 to 31 December 2013 were extracted from the NYISO databases. This period is interesting because it contains a ten-year consumption record peak of 33,956 MW at the end of a week-long heat wave in July 2013, which was used for testing the forecasting results on prolonged temperature extremes. In the data preparation, some errors in the databases were removed and missing values interpolated. A considerable number of outliers were also found, and the most obvious were treated as missing values. For each zone, we prepared the data set with the average hourly load values and daily maximum/minimum temperature realizations and forecasts. The impact of the special days was taken into account by incorporating New Year Eve (31 December, 1 January), Easter (Good Friday, Easter Sunday), and several major public holidays, i.e., Memorial Day, Independence Day, and Columbus Day. Holidays and DST dates for the selected period were downloaded from the Internet. All analysis and model development were conducted in the RStudio environment, using the mgcv package.

### 4.2. Residual Analysis

**Figure 3:**
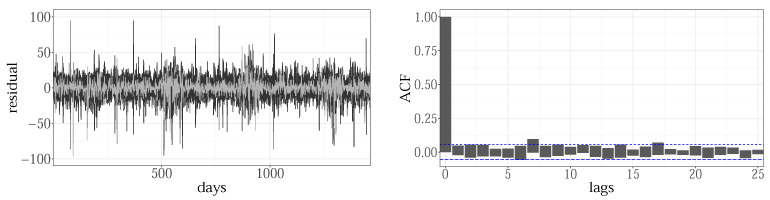
The overlapped residuals’ time plots (**left**) and corresponding autocorrelation (**right**) of hourly time series, for GENESE zone.

For hourly load values, for eleven zones, the NYISO set contains 24 × 11 = 264 load time series. Each is modeled with the GAM terms from Table 2, selected and estimated using PIRLS/REML/DP. In order to investigate whether the proposed GAM dictionary has sufficient capacity to describe them all, a residual analysis was conducted. Four years of load and temperature data, covering 2009 up to 2012, were used for training the models. First, the residuals of individual models were tested for any trend, level, or correlation (left). The results are exemplified on hourly loads for the GENESE zone, in Figure 3: the overlapped residual time plots for all hours (left) and corresponding autocorrelation plots (right) are given. Except for the remaining outliers, present in all time plots, the residuals are centered at zero with the variation that, for most of the time series, stays constant over the whole period. Higher variance during the summer period was noticed for midday hours (15th hour example highlighted in light grey), especially for more populated zones such as NYC, LONGIL, WEST, and GENESE. This effect is typical for models with exclusively temperature-based weather variables, which may have limitations in explaining the cooling demands during the summer period. No significant correlation (left) was found for any of the models.

**Figure 4:**
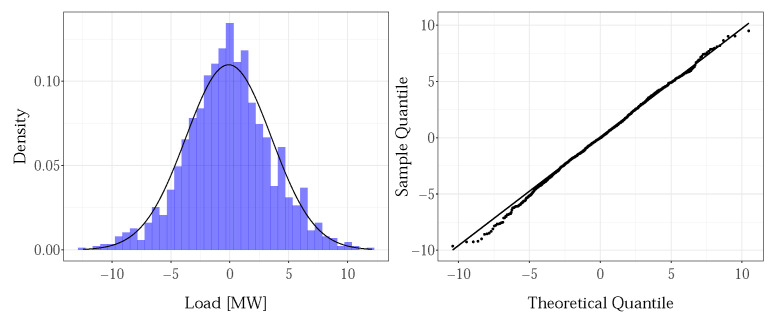
Normality diagnostics of the residuals for the 5th hour, GENESE zone.

**Figure 5:**
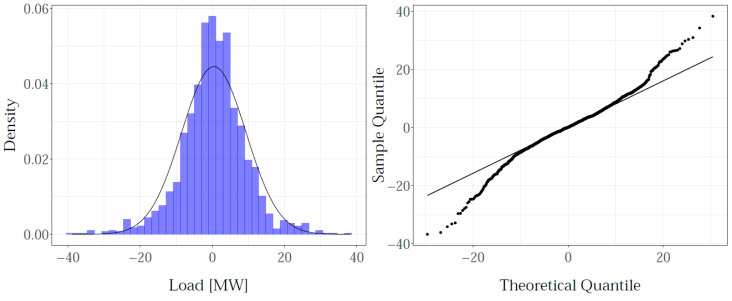
Normality diagnostics of the residuals for the 15th hour, GENESE zone.

A Lilliefors test (5% significance level) was used for checking the normality of the residuals. In order to avoid the influence of outliers, the extreme values were excluded from testing. For most of the residuals, the test rejected the assumption of normality, with the exception of 7% of them, mostly those remaining from the 2nd and 3rd hour models. The histogram and corresponding Q-Q plot indicated that all distributions were symmetric about zero and bell-shaped, without skewness nor kurtosis. Two groups of test outcomes were identified: the residuals whose distribution was close to normal and those for the 13th–16th hours’ models, where the normality assumption was violated. As an illustration, Figure 4 and Figure 5 give the results for the 5th and 15th hours, for the GENESE zone. For both, the Lilliefors test rejected the normality assumptions. For the fifth hour, the descriptive statistics indicates a distribution very close to normal, which was a typical result for most of the residuals. However, for the 15th hour, the deviation from normality is evident and indicates a mixture of distributions. Further analysis revealed two overlapping distributions, for the summer period and the rest of the year.

**Figure 6:**
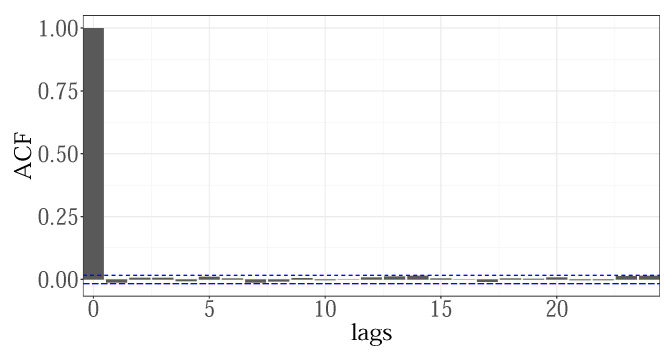
The autocorrelation of the overall residual for GENESE zone.

The overall residual for the GENESE zone, given in the Figure 6, obtained by combining the individual hourly residuals, showed no signs of autocorrelation, which was valid for all zones. This indicates that by including the loads from the two previous hours, $f_{8}\left(L_{h-1}\right)$, $f_{9}\left(L_{h-2}\right)$, the between-hours correlations are fully captured.

**Figure 7:**
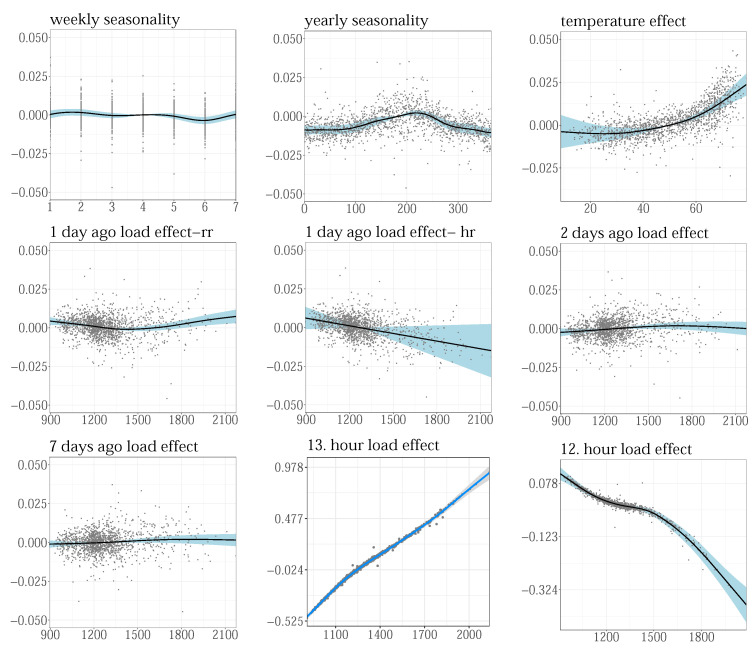
Partial residual for the 14th hour for GENESE zone.

The partial residuals from the estimated smoothers were visually inspected to test if their basis dimensions were properly chosen. As an illustration, Figure 7 shows the partial effects of some terms selected by the VS procedure, for the 14th hour GENESE zone. Weekly and yearly patterns, $f_{w}\left(week\right)$ and $f_{y}\left(year\right)$, maximal temperature for the current day, $f(T_{mx}^{d})$, previous day load on regular days, DT1x$f(L_{14}^{d-1})$, and holidays, DT2x$f(L_{14}^{d-1})$, two days ago loads, $f(L_{14}^{d-2})$, and loads from the previous week, $f(L_{14}^{d-7})$ and from the previous two hours $f(L_{13}^{d})$, $f(L_{12}^{d})$ were selected as significant. The plots were generated using the visreg R function, representing relative changes that particular smoother produced, for the values of the input variable on the *x*-axis. It shows the estimated mean of the smoother (black), the 95% confidence interval (blue), and partial residuals. The residuals, well scattered over correctly modeled means, with no systematic deviation, indicate properly dimensioned smoothers.

The results of the residual analysis, represented for a single zone above, were typical for all NYISO time series. They showed that, for the available data, the proposed methodology based on the GAM dictionary and the implemented VS has the capacity to capture the main features of all loads from the dataset.

### 4.3. Some Comments on VS Results

As explained, the embedded variable selection mechanism selects the important load features, while estimating the model. The VS results were approached by checking the *p*-values (<0.05) associated with the model terms, using the dedicated mgcv function. Given a large number of treated time series, some findings for all zones and hours are discussed. The weekly and yearly profiles and impact of the loads from the previous hours are highly significant for, almost, all load time series. The time series are predominantly determined, either by basic load components (seasonalities, their interactions and trend) or by recent loads, while some are temperature-driven. Night hours, for example, are described with the yearly and weekly profiles and their variations, with a sporadically significant previous day load and temperature. For morning (7th–9th) and afternoon (14th–17th) hours, the recent load history effect converges to its minimal structure, which accounts for the previous day and previous week values, while for diurnal non-working hours, the whole recent effect is significant. Regarding the temperatures, generally, the actual day values (especially the maximum) are of the most importance, while the previous two days’ temperatures and variations over the week and year affect some hours and zones. The midday hours are mostly temperature-driven. The special days are more significant for the diurnal hours, starting from the seventh hour. From the VS results, for small zones, just separating holiday after regular from regular after regular days proved to be sufficient, while for the NYC zone, for example, all four categories were of use for describing the holiday effect. The changes due to daylight saving (DS) mostly affects the 6th–10th hours in the morning and 17th–18th hours in the evening, i.e., the time around the sunrise/sunset.

### 4.4. Predictive Performances

The predictive performances of the generated load models were evaluated with the one-hour-ahead forecasting results, for selected test data. For a specific hour, the load was forecasted by the procedure from Section 2.3, using gam, defined by the dictionary from Table 2 and trained on historical loads for that hour and corresponding temperature realizations. The forecasts were generated using real temperature values. Four years of data were chosen as the optimal training set size, assumed to be sufficient to capture the dynamics of the implemented load features. Thus, loads and temperatures for the first four years, i.e., from 1 January 2009 to 31 December 2012, initially, went to models’ training, providing the first forecast and its evaluation for the first hour in 2013. The four-year training data window and the procedure moved further, throughout 2013. The single model estimation (with over 400 coefficients to be calculated) and forecast generation took several seconds on an AMD Ryzen 5 3500U, 2.10 GHz, 8.00 GB RAM machine.

The quality of the prediction was measured with the mean absolute percentage error (MAPE) and mean absolute error (MAE), which, for *M* observations, are defined in Equations (10) and (11), for the actual load value, $L_{h}$, and its forecast, ${\hat{L}}_{h}$:

(10) $$\mathrm{MAPE}=\frac{100}{M}\sum_{h=1}^{M}\left|\frac{L_{h}-{\hat{L}}_{h}}{L_{h}}\right|$$

(11) $$\mathrm{MAE}=\frac{1}{M}\sum_{h=1}^{M}\left|L_{h}-{\hat{L}}_{h}\right|$$

The mean MAPE/MAE values for 2013, for each zone, are given in Table 3.

**Figure 8:**
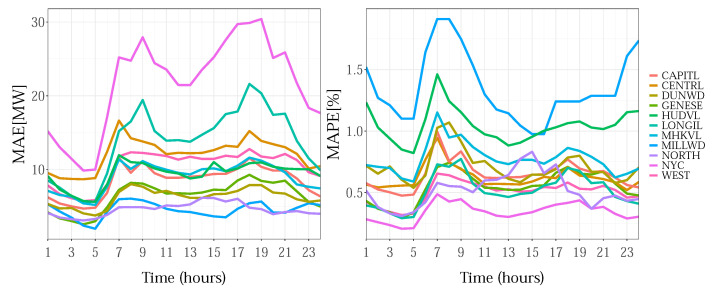
Hourly MAE/MAPE results for NYISO zones for 2013.

The MAPE/MAE distribution by hour for each zone is given in Figure 8. The larger errors around the daily peaks, especially those for the morning hours, are visible.

**Figure 9:**
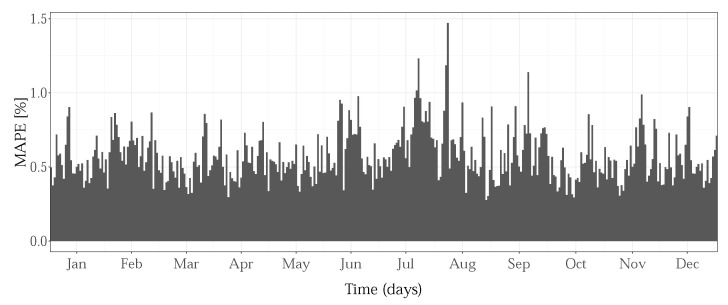
Mean daily MAPE for GENESE zone, for 2013.

To gain insight into the performances of zonal models over time, we observed the mean daily MAPE for the entire test period, for each zone. The bar graph from Figure 9 shows an example, only, for GENESE, as the results are qualitatively the same for the other zones. Higher error values for the critical period around the consumption peak in July were obtained.

### 4.5. Forecasting Results

As a basic STLF functionality, the real forecasting scenario for the next 24-h time horizon was observed. Accordingly, the forecasting was initiated each day at 00 h, preceded by an update of hourly submodels with the most actual available data history, up to the end of the previous day. A step ahead forecast of each submodel, based on available temperatures forecasts for the coming day, covers the next 24 h with the load prognosis. Since the model also incorporates the load values from previous hours as inputs, the forecasts for 2. …24 h were obtained, recursively, using previous forecasts for 1. …23 h. The same procedure was run, also, for real temperature values (instead of forecasts) in order to gain insight into the error caused by uncertainty of temperature forecasts.

**Figure 10:**
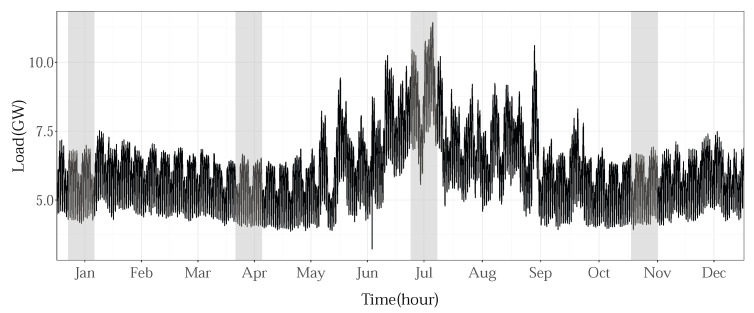
Selected time intervals for testing (NYC zone).

Two-week time periods for each season, avoiding public holidays, were selected for testing and representing the results: 7 January 2013–20 January 2013 (winter), 6 April 2013–19 April 2013 (spring), 7 July 2013–20 July 2013 (summer), and 2 November 2013–15 November 2013 (fall). In Figure 10, which represents the hourly loads for 2013, for the NYC zone, the chosen time periods are marked with the shaded intervals. As is obvious, the period with maximum consumption in 2013 was also included for testing.

The results of the zonal MAPE (MAE) over the corresponding time periods, for forecasted (f) and real (r) temperature values, are given in Table 4. The expected MAPE values for each zone are given in the last column.

Although no strict comparative study was conducted, it was found that the MAPE values, obtained for 24 h ahead, are competitive with some recently reported for the NYISO data set, [38,39]. The minimum accuracy was achieved for the MILLWD zone. This is the zone with the lowest average consumption of about 300 MW, and a standard load variation results in higher MAPE error. The MAPE errors are higher during the summer cooling period than for other seasons, less pronounced for the low populated NORTH and small MILLWD zones, while noticeable for highly urban, big regions. In general, the residual tests and overall forecasting results indicate that further improvements of the proposed model are needed to overcome limitations in explaining the summer rise in consumption caused by the significant number of air conditioners. This is in accordance with the results from [36], where it was reported that, for U.S. regions, the predictions based only on the air temperature tend to underestimate the load during the summer period. The perceived heat during the prolonged summer temperature extreme causes a faster-than-predictable increase in the load, not explainable only with the temperature indices. The additional weather variables, the near-surface humidity, recommended in [36], can be included in the model with the appropriate GAM term, added to the dictionary.

To test the special day effect, forecasts for selected holiday dates were compared with those produced by a model treating all days as regular. Finally, the DT variable was removed from the model. The obtained MAPE errors are given in Table 5 for the model with all holidays accounted for (in) and excluded (out).

**Figure 11:**
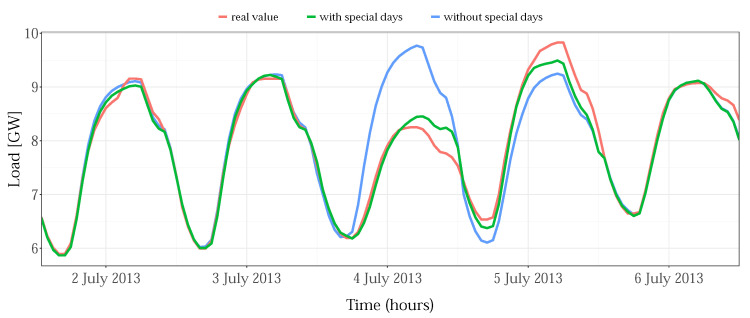
Special day effect for the example of Independence Day (4 July 2013), for NYC zone.

The proposed approach to the treatment of special days does not take into account the specifics of individual holidays and models the hourly reduction in consumption, averaged for all them. According to the values from Table 5, for most public holidays, which are days off for most of the population, schools, and businesses, such as Memorial Day or Independence Day, the MAPE error is reduced by more than 50%. For some zones/holidays, this reduction is even close to the accuracy of a regular day. As an illustration, the case of Independence Day for the NYC zone is shown in Figure 11: without treated special days, the forecast results give 10.8% MAPE (blue to red line error), while including them in the model, the MAPE decreased to 2.0% for that day (green to red line).

For Columbus Day, for example, the MAPE results without special day treatments are not as bad, and consequently, the improvements are not as pronounced. It is possible that it is only partially a non-working day and acts similar to a regular day. Easter, when considered as a regular day, also has a slightly lower MAPE, as it falls on Sunday, and lower consumption has been captured with week seasonality. However, NY Eve and Easter create a more complex holiday effect, which requires more attention. In general, taking into account some specifics of individual holidays or, at least, categories of holidays would improve the accuracy of these days’ predictions.

The DST-induced load changes are most noticeable in the period immediately after its implementation. Therefore, the first week after the spring clock change (covering the period from 11–17 March) was selected as a reference for evaluating the incorporated DST effect. As in the case of special days, the results of forecasting for these dates between the full model and those with the excluded DST effect, i.e., without the DSx$f_{y}\left(year\right)$ and DSx$f_{w}\left(week\right)$ terms, were compared. The average MAPE results for this week and for each zone, given in Table 6, indicate an improvement in forecasting accuracy for the period mostly affected by the DST effect.

**Figure 12:**
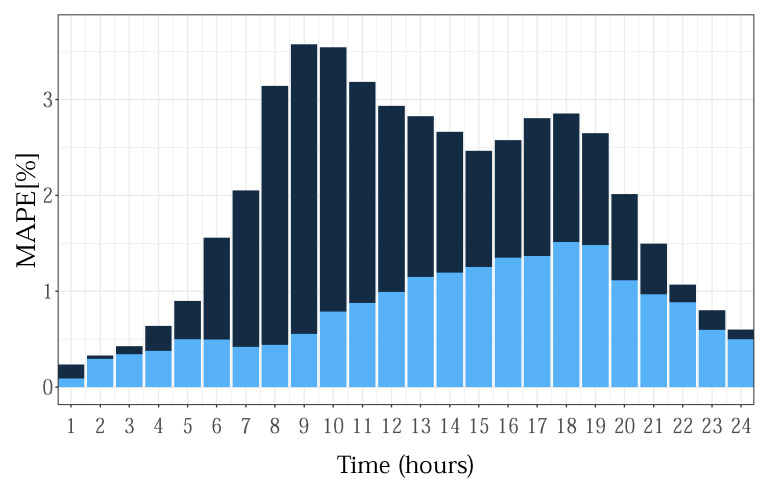
Average MAPE result for each hour in a day for the week after DST (NYC zone).

Since it is related to consumers’ habits, the DST effect, as well as its reduction are more pronounced for highly populated zones. In Figure 12, the average MAPE error for each hour in the day, over the post-DST week, for the NYC zone is given.

The daylight saving effect is based on the clock change dates, with DST built into the annual and weekly profiles of a single load. However, when daylight information is available, as is the case in [37], it can be used to construct appropriate GAM terms for a more general approach to the DST effect.

## 5. Conclusions

This paper presents a methodology for forecasting the multiple loads in the grid, using the generalized additive model (GAM). It is based on an application-specific dictionary of standardized GAM terms and a procedure for its dynamical adaptation and forecast generation for individual loads.

The main advantage of the proposed approach is that it reduces the complex problem of developing multi-load forecasting to a simpler task of GAM dictionary construction. GAM terms are spline-based elements, able to capture complex dependencies, without significant prior assumptions nor critical hyperparameters involved, which can be standardized for a specific load forecasting application, defined with the aggregation level, forecasting horizon, and available data. The selection of terms and dictionary adaptation to a specific load was performed by the shrinkage-type variable selection. In this work, an example of a dictionary, constructed for loads for eleven NYISO zones, was presented, prepared for a 24-h load forecasting functionality, using available historical values for loads and temperatures. Residual analysis and one-hour-ahead prediction results confirmed the generalizability of the proposed methodology and its ability to cope with the heterogeneity of the NYISO load profiles. A case study for 24-hour-ahead forecasting, in real conditions, produced results competitive with some recently reported and demonstrated the ability of the generated models to cope with the characteristic load features, such as the special day effect, daylight saving effect, etc. However, the NYISO dictionary needs to be updated with additional weather variables, to improve the modeling of the weather-induced load during summer. The next step is to evaluate the approach for different consumer coverage, primarily for buildings and smaller consumer aggregations, which are of particular practical importance in smart grids.

Another advantage of the presented methodology is the concept of dynamic variable selection, addressed to the shrinkage embedded into the fast estimation procedure, which enables fast, dynamic remodeling with each new datum. It represents an adaptive potential of the load forecasting functionality in the presence of various uncertainties in the grid, which has yet to be evaluated.

## Figures and Tables

**Table 1:** NYISO zones’ average consumption and number of residents.

Zone	Zone ID	Average Load (MW)	Population [×$10^{3}$]
West	WEST	1790	1532
Genese	GENESE	1140	1003
Central	CENTRL	1850	1384
Capital	CAPITL	1330	1215
Millwood	MILLWD	330	190
Dunwoodie	DUNWD	670	760
New York City	N.Y.C.	6120	8186
Long Island	LONGIL	2540	2835
Mohawk Valley	MHKVL	910	891
Hudson Valley	HUDVL	1150	1372
North	NORTH	540	82

**Table 2:** Dictionary of GAM load terms.

	Model Term	Feature
0	$lnL_{t}$	the load at time instance *t*, in a day *d*
1	$f_{t}\left(trend\right)$	TPRS spline for long-term trend
2	$f_{y}\left(year\right)$	cyclic CRS for an annual cycle with 12 knots
3	$f_{w}\left(week\right)$	cyclic CRS with 7 knots
4	$f_{w,y}\left(week,year\right)$	week and year smooth interaction term
5	$DSf_{y}\left(year\right)$	daylight saving/yearly cycle interaction
6	$DSf_{w}\left(week\right)$	daylight saving/weekly cycle interaction
7–12	$f(T_{x}^{y})$	TPRS spline for each combination of temperature type, *x*, and day, *y*, where *x* is the maximal/minimal temperature, for *y*, the current and previous two days, *d*, d−1, d−2
13–15	$f_{T_{mx}^{y},T_{mn}^{y}}\left(T_{mx}^{y},T_{mn}^{y}\right)$	maximal/minimal temperature interaction term for *y* current and previous two days, *d*, d−1, d−2
16–21	$f_{T_{x}^{y},week}\left(T_{x}^{y},week\right)$	week/maximal temperature interaction term for *x*, the maximal/minimal temperature, for *y*, the current and previous two days, *d*, d−1, d−2
22–27	$f_{T_{x}^{y},year}\left(T_{x}^{y},year\right)$	year/maximal temperature interaction term for *x*, the maximal/minimal temperature, for *y*, the current and previous two days, *d*, d−1, d−2
28	DT	categorical variable for day type (realized as a factor variable)
29	$DTf_{1}\left(L_{t}^{d-1}\right)$	day type/previous day load interaction
30–35	$f_{2}(L_{t}^{d-2}\ldots f_{7}\left(L_{t}^{d-7}\right)$	TPRS splines for the same time, previous −2 to −7 days
36–37	$f_{8}\left(L_{t-1}\right),f_{9}\left(L_{t-2}\right)$	TPRS splines for the two previous time instances

**Table 3:** One-hour ahead MAPE/MAE results for all NYISO zones for 2013.

Zone	MAPE (%)	MAE (MW)
WEST	0.50	10.3
GENESE	0.55	6.43
CENTRL	0.62	12.1
CAPITL	0.65	8.5
MILLWD	1.31	4.5
DUNWD	0.71	6.2
N.Y.C.	0.31	21.7
LONGIL	0.52	13.7
NORTH	0.78	9.0
HUDVL	0.98	9.4
MHKVL	0.52	4.6

**Table 4:** MAPE (MAE) results for selected time intervals, for each NYISO zone.

Zone	f/r	Winter	Spring	Summer	Fall	Averaged
WEST	f	1.22 (22.87)	1.50 (27.29)	2.31 (49.87)	1.34 (25.42)	**1.59 (31.36)**
	r	1.18 (22.28)	1.34 (23.41)	2.02 (42.55)	1.31 (24.90)	**1.46 (28.29)**
GENESE	f	1.40 (20.05)	1.46 (16.35)	2.06 (30.87)	1.15 (12.53)	**1.52 (19.95)**
	r	1.32 (19.01)	1.34 (14.71)	1.81 (26.43)	1.07 (12.14)	**1.39 (18.07)**
CENTRL	f	1.42 (28.32)	1.28 (22.28)	1.93 (43.91)	1.46 (28.61)	**1.52 (30.78)**
	r	1.32 (25.71)	1.20 (21.21)	1.85 (42.81)	1.42 (27.63)	**1.44 (29.34)**
CAPITL	f	1.53 (20.84)	1.69 (23.82)	2.01 (35.79)	1.62 (21.92)	**1.71 (25.59)**
	r	1.43 (19.38)	1.63 (20.62)	1.94 (34.76)	1.55 (20.81)	**1.64 (23.89)**
MILLWD	f	2.75 (9.36)	2.92 (9,99)	2.97 (15.58)	2.94 (9.33)	**2.89 (11.06)**
	r	2.35 (7.91)	2.73 (8.29)	2.88 (14.89)	2.73 (9.29)	**2.67 (10.01)**
DUNWD	f	2.01 (14.51)	2.00 (13.58)	3.12 (36.87)	1.51 (10.32)	**2.16 (18.82)**
	r	1.80 (12.26)	1.76 (12.37)	2.81 (36,87)	1.36 (9.14)	**1.93 (17.66)**
NYC	f	1.10 (64.60)	1.31 (74.62)	2.42 (217.54)	0.83 (54.71)	**1.42 (102.87)**
	r	0.90 (52.82)	1.03 (57.73)	2.06 (193.29)	0.79 (49.52)	**1.20 (88.34)**
LONGIL	f	1.77 (45,94)	1.72 (40.22)	2.72 (112.38)	1.79 (41.62)	**2.00 (60.04)**
	r	1.73 (45.73)	1.61 (36.01)	2.67 (108.23)	1.55 (35.92)	**1.89 (56.47)**
NORTH	f	1.17 (9.53)	1.01 (7.81)	1.15 (8.83)	1.51 (11.83)	**1.21 (9.50)**
	r	0.98 (8.00)	0.92 (7.06)	1.09 (8.33)	1.38 (10.64)	**1.09 (8.51)**
HUDVL	f	1.65 (18.77)	1.68 (17.91)	2.82 (47.90)	1.64 (18.3)	**1.88 (25.72)**
	r	1.60 (18.10)	1.53 (15.82)	2.69 (42.05)	1.42 (15.7)	**1.81 (22.92)**
MHKVL	f	1.82 (20.21)	2.28 (25.41)	2.68 (30.67)	2.06 (19.54)	**2.21 (23.96)**
	r	1.76 (19.03)	2.02(20.12)	2.63 (29.60)	2.00 (18.85)	**2.01 (21.90)**

**Table 5:** MAPE results for special days for each NYISO zone.

Zone	In/Out	1 January	Easter Sunday	Memorial	Independence	Columbus
WEST	in	2.91	0.94	2.23	2.72	2.03
	out	7.94	5.72	10.50	8.30	2.15
GENESE	in	3.52	1.51	2.15	3.82	1.92
	out	11.59	8.72	12.50	10.10	2.14
CENTRL	in	3.62	1.58	1.74	3.34	1.24
	out	5.30	6.64	8.82	7.23	1.38
CAPITL	in	4.92	2.24	3.25	3.13	1.52
	out	10.12	3.82	11.81	7.94	1.77
MILLWD	in	2.54	3.23	3.92	2.53	2.22
	out	10.81	3.53	11.26	3.72	2.27
DUNWD	in	3.72	2.53	2.10	2.31	2.43
	out	10.12	2.95	9.74	9.34	2.55
N.Y.C.	in	3.40	1.33	1.32	2.01	3.21
	out	11.23	5.97	9.90	10.80	3.39
LONGIL	in	2.84	3.17	3.41	3.32	3.50
	out	9.16	3.95	10.92	5.27	3.67
NORTH	in	1.72	0.88	1.24	0.92	0.91
	out	1.83	1.84	2.63	2.32	1.01
HUDVL	in	3.72	1.13	4.62	1.53	1.63
	out	8.73	5.94	7.81	3.35	1.73
MHKVL	in	3.21	2.10	3.26	1.92	2.21
	out	9.97	4.72	14.62	4.56	2.34

**Table 6:** DST effect for selected time intervals, for all NYISO zones.

Zone	Without DST	With DST
WEST	1.58	1.28
GENESE	1.84	1.65
CENTRL	2.32	2.21
CAPITL	1.94	1.66
MILLWD	4.50	4.23
DUNWD	2.05	1.78
N.Y.C.	1.97	0.85
LONGIL	1.92	1.58
NORTH	2.16	1.38
HUDVL	2.68	2.64
MHKVL	1.88	1.85

## Data Availability

https://www.nyiso.com/load-data (accessed on 23 June 2022).
